# An Approach to Solid-State Electrical Double Layer Capacitors Fabricated with Graphene Oxide-Doped, Ionic Liquid-Based Solid Copolymer Electrolytes

**DOI:** 10.3390/ma9060450

**Published:** 2016-06-06

**Authors:** N. F. A. Fattah, H. M. Ng, Y. K. Mahipal, Arshid Numan, S. Ramesh, K. Ramesh

**Affiliations:** Centre for Ionics Universiti Malaya, Department of Physics, Faculty of Science, University of Malaya, Kuala Lumpur 50603, Malaysia; faiqahfattah@gmail.com (N.F.A.F.); nghonming@hotmail.com (H.M.N.); ykmahipal@gmail.com (Y.K.M.); numan.arshed@yahoo.com (A.N.); ramesh@um.edu.my (K.R.)

**Keywords:** copolymer, graphene oxide, electrical double layer capacitor, ionic liquid

## Abstract

Solid polymer electrolyte (SPE) composed of semi-crystalline poly (vinylidene fluoride-hexafluoropropylene) [P(VdF-HFP)] copolymer, 1-ethyl-3-methylimidazolium bis (trifluoromethyl sulphonyl) imide [EMI-BTI] and graphene oxide (GO) was prepared and its performance evaluated. The effects of GO nano-filler were investigated in terms of enhancement in ionic conductivity along with the electrochemical properties of its electrical double layer capacitors (EDLC). The GO-doped SPE shows improvement in ionic conductivity compared to the P(VdF-HFP)-[EMI-BTI] SPE system due to the existence of the abundant oxygen-containing functional group in GO that assists in the improvement of the ion mobility in the polymer matrix. The complexation of the materials in the SPE is confirmed in X-ray diffraction (XRD) and thermogravimetric analysis (TGA) studies. The electrochemical performance of EDLC fabricated with GO-doped SPE is examined using cyclic voltammetry and charge–discharge techniques. The maximum specific capacitance obtained is 29.6 F∙g^−1^, which is observed at a scan rate of 3 mV/s in 6 wt % GO-doped, SPE-based EDLC. It also has excellent cyclic retention as it is able keep the performance of the EDLC at 94% even after 3000 cycles. These results suggest GO doped SPE plays a significant role in energy storage application.

## 1. Introduction

Electronic devices are becoming essential belongings for people around the world in their daily life. It is something that is carried along almost everywhere at all times. Thus, the need for these devices to be lightweight, thin and safe is extremely high. The conventional liquid electrolyte batteries used in these electronic devices now are mostly bulky and heavy and could cause safety issues due to the risk of harmful liquid leakages [1]. Recent research has shown tremendous advancements in an alternative to liquid electrolyte batteries: solid polymer electrolytes (SPE). Their ability to form thin films, light weight, flexibility and reasonable ionic conductivity are among the useful benefits of using solid polymer electrolyte [2].

In previous researches, many different polymers have been used in the application of SPE and most of them are conventional homopolymers such as PVC and PAN [3]. In more recent work, the copolymer has emerged as a potent choice to be used as the host polymer due to the advantages it could provide. For example, in this work, P(VdF-HFP) is used as the host polymer. This copolymer is the result of the copolymerization between poly (vinylidene fluoride) [PVdF] and hexafluoropropylene [HFP]. PVdF has high crystallinity while HFP has high amorphous phase [4]. When copolymerized, the HFP region could drastically reduce the crystallinity and promote more amorphous regions within the copolymer, a desirable quality, which is needed in polymer electrolytes [5]. HFP phase provides higher ionic conduction and a PVdF region provides mechanical stability for the polymer electrolyte film [6].

In this research, 1-ethyl-3-methylimidazolium bis (trifluoromethyl sulphonyl) imide [EMI-BTI] ionic liquid is used. Ionic liquids are molten salts that are in liquid state at room temperature. Ionic liquid was proven to have the ability to enhance the ionic conductivity of the polymer electrolyte due to its high electrical performance properties [7]. In a solid polymer electrolyte system which has no salt added, ionic liquid plays a huge role in providing the mobile ions to allow ion conduction to occur within the polymer matrix of the SPE. Besides having good electrical properties, ionic liquid also well known to have low combustibility as well as excellent chemical and thermal stability which are very much important for the application of SPEs [8].

In addition, nanofiller is usually added into the polymer electrolytes for the purpose of improving the properties of the polymer electrolytes in terms of electrochemical performances, thermal properties, *etc.* [9]. In this research, we focused on the effect of the addition of different weight percentages of graphene oxide (GO), an inorganic nanofiller into the solid polymer electrolyte. GO has a unique property in that it has multiple oxygen-containing functional groups which could enable it to be well dispersed in water or polar solvents and could also improve the mobility of the mobile ions and lead to the improvement of the ion conductions of the polymer electrolytes [10,11].

The GO-doped solid polymer electrolytes were then used with activated carbon electrodes to fabricate EDLC. EDLC has advantages over the usual rechargeable batteries available. It has higher charge energy density, higher rate of charge-discharge and longer cycle performance compared to conventional rechargeable batteries [12,13,14,15]. The electrical charges are stored based on electrostatic interaction in the electric double layer at the interface of electrode or electrolyte [16]. Activated carbon is commonly used as the negative and positive electrodes while the solid polymer films are used as the electrolyte and separator between the cathode and anode. Activated carbon has the characteristics of cost effective, high porosity and high surface area [17]. This project has investigated the electrical performance of GO-doped P(VdF-HFP) solid polymer electrolyte-based EDLC in order to learn more about the compatibility of our SPE for the EDLC application.

## 2. Methodology

### 2.1. Materials

Poly (vinylidene fluoride-hexafluoropropylene) [P(VdF-HFP)], 1-Ethyl-3-methylimidazolium bis (trifluoromethyl sulphonyl) imide [EMI-BTI] and acetone were obtained from Sigma-Aldrich (St. Louis, MO, USA). Meanwhile, graphene oxide (GO) was synthesized with the method according to a study reported by See *et al.* [14]. The sulphuric acid [H_2_SO_4_], potassium permanganate [KMnO_4_] and hydrogen peroxide [H_2_O_2_] that were used for the synthesis of graphene oxide were obtained from Sigma-Aldrich as well.

### 2.2. Synthesis of Graphene Oxide

Graphene oxide was prepared using simplified Hummer’s method from graphite. 400 mL of H_2_SO_4_ and 18 g of KMnO_4_ were used to treat 3 g of graphite flakes oxidatively. The mixture was then stirred for 5 min for the graphite oxide to be formed. In order to secure complete oxidation of graphite, the mixture was then stirred for an additional 3 days. Colour changed during the oxidation process can be observed during the whole stirring process. After that, the change of colour of the mixture to bright yellow after the addition of H_2_O_2_ solution showed that highly oxidized graphite had been obtained. The graphite oxide was then rinsed 3 times with 1 M of HCL and followed by 6-times deionized water to obtained GO gel [18].

### 2.3. Preparation of Graphene Oxide Doped Solid Polymer Electrolyte

**Figure 1:**
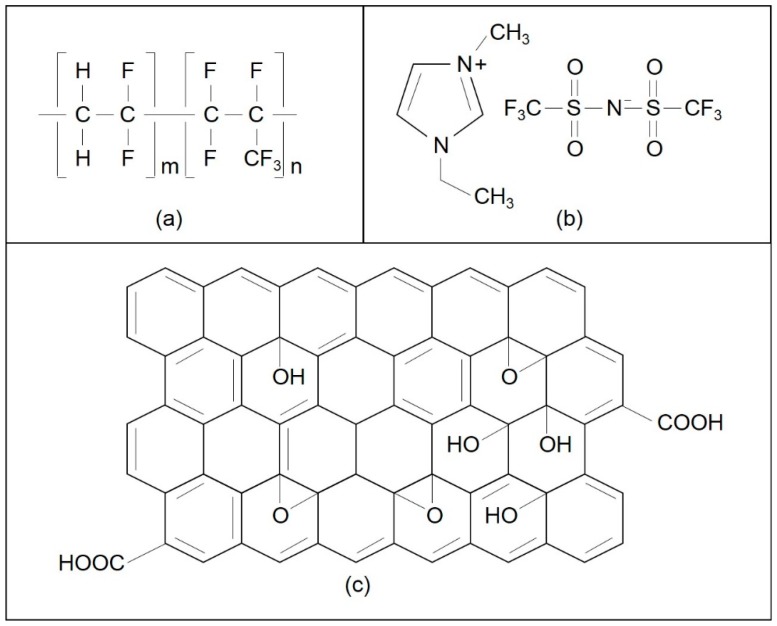
Chemical structure of (**a**) P(VdF-HFP); (**b**) EMI-BTI and (**c**) proposed GO.

P(VdF-HFP)-[EMI-BTI]-based SPE was prepared with a different ratio of the P(VdF-HFP) and EMI-BTI as seen in Table 1. Respective weight P(VdF-HFP) and EMI-BTI was added into 10 mL of acetone in a small container and the mixture was then stirred for 2 h at 40 °C for homogeneity. Then, the homogeneous viscous mixture was cast onto a glass petri-dish. The mixture was then left heated at 80 °C in the oven for 12 h for the thin films to be formed. The sample with the highest optimum conducting composition (OCC) was then added with graphene oxide. A series of P(VdF-HFP)-[EMI-BTI]-GO SPEs (2 wt % to 8 wt % of GO) with the composition as seen in Table 1 were prepared by the same solution casting method as mentioned above. Figure 1 shows the chemical structure of the used materials.

### 2.4. Fabrication of the Electrode

The electrode was fabricated with ratio of 75 wt % activated carbon with a particle size between 5–20 µm and surface area between 1800 and 2000 m^2^/g, 10 wt % Poly (vinylidene fluoride) (PVdF) binder and 15 wt % carbon black (Super P). This mixture was mixed in 1-Methyl-2-pyrrolidone (NMP) solvent to produce a slurry mixture. The slurry using drop casting method was coated onto 1 cm^2^ stainless steel foil. The foil was rubbed with different grades of emery paper rinsed with hydrochloric acid (HCl), deionized water and immersed in acetone before coating. The electrodes were dried in an oven at 100 °C overnight. The difference of mass of foil before and after coating was calculated in order to obtain the mass of loading of carbon mixture [19].

### 2.5. Characterization Techniques

The solid polymer electrolyte samples were subjected to EIS characterization using Hioki 3532-50 LCR-Hi tester (Nagano, Japan) in the frequency range of 50–5,000,000 Hz at room temperature. The ionic conductivities σ (S∙cm^−1^) of all the samples were then calculated by using the following equation [20]:

σ = *t*/(*R_b_* × *A*) (1)

where *R_b_* is the bulk resistance of the sample which was obtained from the Nyquist impedance plot, *A* is the area of the contact of the electrodes with the electrolytes in cm^2^ and *t* is the thickness of the sample in cm. Temperature-dependent ionic conductivity was measured at temperatures that varied from 30 °C to 60 °C. 

FTIR studies of all the SPE samples were conducted using a Thermoscientific Nicolet iS10 FTIR spectrometer (Thermo Fisher Scientific, Waltham, MA, USA) over the range of 650–4000 cm^−1^. A Philips X-ray diffractometer with Cu Kα radiation (PANalytical, Almelo, The Netherlands) was used to identify the crystallinity and amorphous phases of the samples. The 2θ angle is varied from 0° to 55°. Thermal properties were studied using thermogravimetric analysis TGA Q500 V20.13 Build 39 (TA, Instruments, New Castle, DE, USA) with a temperature up to 800 °C.

### 2.6. Fabrication and the Electrochemical Measurements of EDLC

The EDLC cells were fabricated using the configuration: AC/GO-doped SPE/AC. The cells were then characterized using cyclic voltammetry (CV), galvanostatic charge-discharge (CCD) and electrochemical impedance spectroscopy (EIS) with AC amplitude of 10 mV from 0.01 to 100,000 Hz frequency range.

## 3. Results and Discussion

### 3.1. EIS Studies of Solid Polymer Electrolytes

**Figure 2:**
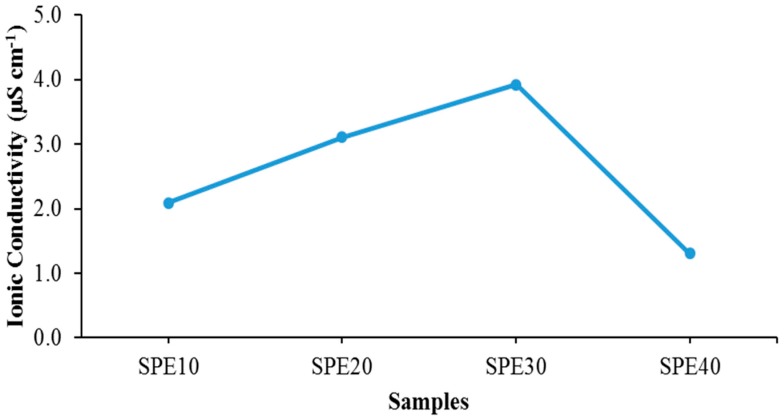
Ionic conductivity for the SPEs with different ratio of EMI-BTI and P(VdF-HFP) at room temperature.

Figure 2 and Table 2 shows the ionic conductivity for PVdF-HFP-[EMI-BTI] based SPEs samples (SPE10–SPE40). Sample SPE30 shows the highest ionic conductivity at 3.92 μS∙cm^−1^ at room temperature. This sample was then chosen to be the optimum conducting composition (OCC) sample for this system and was then added with GO for further investigation of the effects of the addition of GO. The increase of the ionic conductivity up to 30 wt % of the EMI-BTI added is due to the increasing number of mobile ions [21] and also the plasticizing effect from the EMI-BTI ionic liquid [22,23]. However, there is a decrease in ionic conductivity that could be observed when 40 wt % of EMI-BTI added into the system. This is due to the agglomeration of the excess ions from the EMI-BTI which causes the mobile ions to form neutral pairs, and this could lead to a decrease in ionic mobility of the SPEs [24].

**Figure 3:**
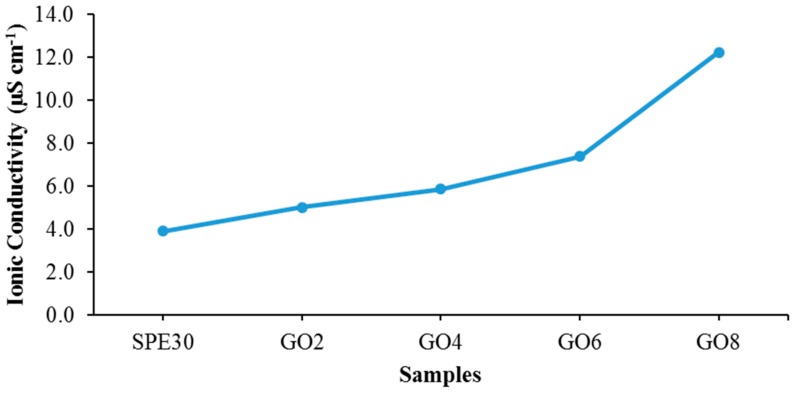
Ionic conductivity of PVdF-HFP-[EMI-BTI]-based SPE samples added with different weight percentages of GO at room temperature.

The value of ionic conductivity for SPE samples with different weight percentages of GO is plotted in Figure 3 and shown in Table 2. The highest value of conductivity observed was at 12.25 μS·cm^−1^ at 8 wt % of GO added. A significant increase in ionic conductivity was observed upon the addition of the graphene oxide nano-filler. The increase of ionic conductivity, after the addition of graphene oxide, is due to the nature of graphene oxide that has a lot of oxygen-containing functional groups such as carboxylic acid, epoxy and hydroxyl [25] which can improve charge carrier mobility and promote ionic conductivity [26]. Besides, by having a huge number of functional groups, it also helps GO to be able to dissolve much more efficiently in various types of solvents, and in this case, acetone compared to other nanofillers such as the conventional silica (SiO_2_). This causes GO to be able to homogeneously distribute in the SPEs, and could facilitate the mobility of mobile charge ions and leads to increase in ionic conductivity [27]. GO could also act as nanofiller where it can help to promote more conducting pathways so that the mobile ions move easily in the polymer matrix. As seen in Figure 3, the studies of the addition of GO into the P(VdF-HFP) based SPE were halted at 8 wt % of the GO added. Supposedly, further studies are needed for the addition of more GO into the system. However, in this work, the film for 10 wt % of GO doped SPE sample was too brittle rendering further characterization impossible.

### 3.2. Ion Conducting Mechanism

**Figure 4:**
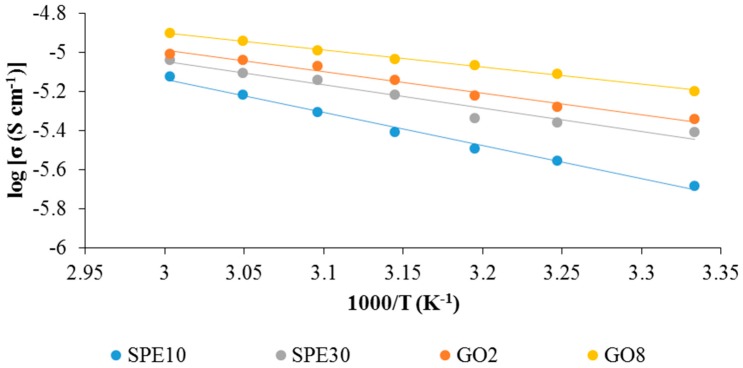
Arrhenius plots for the conductivity of the PVdF-HFP-[EMI-BTI]-based SPE samples.

Figure 4 shows the temperature dependence ionic conductivity for sample SPE10, SPE30, GO2 and GO8. The temperature dependence studies were done in the range of 303 K to 333 K. Temperature dependence of ionic conductivity was analyzed in order to study the ionic conduction mechanism of the SPE. It can be seen from the figure that the results presented regression lines close to unity (*R* ~ 1) for all of the samples. The linearity of these lines shows that the SPE samples were obeying Arrhenius behaviour which can also be expressed as:

σ = σ_0_ exp (−*E*_a_/k*T*) (2)

By obeying the Arrhenius behaviour, the ion conduction mechanism of all of the SPE samples was concluded to be the hopping of the ions from one site to another neighbouring site in the polymer matrix. From the figures, it can also be observed that there was an increase in ionic conductivity with the increase of temperature. This is due to the higher segmental motion of polymer chain in the amorphous region upon addition of heat. On the other hand, the activation energy (*E*_a_) of the SPEs was obtained from Figure 4 and was tabulated in Table 2. It can be seen that sample GO8 has the lowest activation energy and this is in agreement with the results in ionic conductivity where normally the sample with the higher ionic conductivity would have lowered the activation energy [28]. The *E*_a_ values were also found to be comparable to other articles [29,30].

### 3.3. FTIR Measurements of Solid Polymer Electrolytes

**Figure 5:**
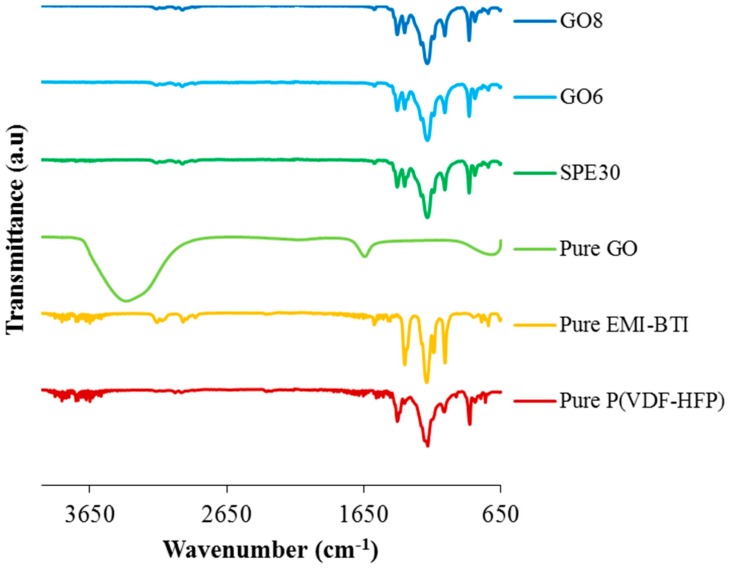
FTIR spectra of P(VdF-HFP), EMI-BTI, GO, sample SPE30, GO6 and G8.

In order to understand the interaction between the polymer, ionic liquid and GO, the IR spectra of pure materials, SPE samples with and without GO added were presented in Figure 5. Some of the important band assignments has already been reported for pure P(VdF-HFP) and EMI-BTI and the band assignments are listed in Table 3 [31,32]. The similar FTIR spectrum of GO has been reported by Cao *et al.*, where the GO spectrum shows the presence of a different oxygen functional group in the GO. The peaks at 3383 cm^−1^ and 1645 cm^−1^ were assigned to O-H stretching vibration and stretching vibrations from C=O or COOH, respectively [33]. 

Initially, the addition of EMI-BTI into SPE shifted the peaks at 796 cm^−1^, 1182 cm^−1^, and 1404 cm^−1^ of pure P(VdF-HFP) to 791 cm^−1^, 1169 cm^−1^, and 1400 cm^−1^, respectively, in SPE30. The peak at 1202 cm^−1^ of P(VdF-HFP) was nowhere to be found in SPE30. This phenomenon indicates that there were interactions between the mobile ions from the EMI-BTI ionic liquid and the polymer matrix. Fluorine inside the P(VdF-HFP) is an electron donating atom and this is where the EMI^+^ cation would form a dative bond with the fluorine to form P(VdF-HFP)-ionic liquid complexes [34]. On the other hand, upon the addition of GO, the peaks which were initially found in the spectrum of GO were nowhere to be found in the spectra of SPE with GO (GO6 and GO8). Due to the disappearance of these peaks, it was believed that the functional groups of the GO also formed a complexation with the solid polymer electrolytes.

### 3.4. X-ray Diffraction Studies

**Figure 6:**
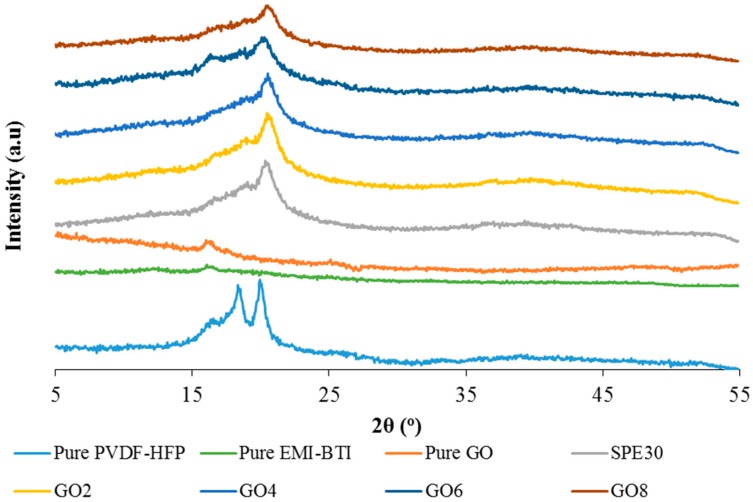
XRD pattern for pure P(VdF-HFP), pure EMI-BTI, pure GO, SPE30 and different weight percentage of GO-doped PVdF-HFP-[EMI-BTI]-based SPEs samples.

Figure 6 shows the XRD pattern for pure PVdF-HFP, pure EMI-BTI, pure GO, SPE30 and the GO-doped P(VdF-HFP)-(EMI-BTI)-based SPEs samples. As seen in the XRD pattern of the pure PVdF-HFP, the high intensity peak at around 2θ = 17° and 22.4° shows the highly crystalline nature of the pure PVdF-HFP. However, upon the addition of the ionic liquid as seen in SPE30, the intensity of the peak at 2θ = 22.4° was found to be decreased and the peak at 2θ = 17° was found to be disappeared from the XRD pattern.

This shows that the addition of the ionic liquid decreased the crystallinity of the backbone of the PVdF-HFP and increased the amorphous region of the SPE samples [35]. This is in agreement with what has been found in ionic conductivity studies. Furthermore, the changes in the intensity and the disappearance of peaks show that there is some complexation occurring in the polymer matrix of the SPE. The addition of GO was also found to decrease the intensity of the peak at 2θ = 23° as observed in Figure 6. The intensity of the peak further decreased as more GO was added into the SPE. This proves that the addition of GO increases the amorphous region of the SPE samples, which could assist in improving the mobility of the mobile charge ions in the SPEs and leads to an increase in the ionic conductivity as observed in the previous studies [36].

### 3.5. Thermogravimetric Analysis (TGA)

**Figure 7:**
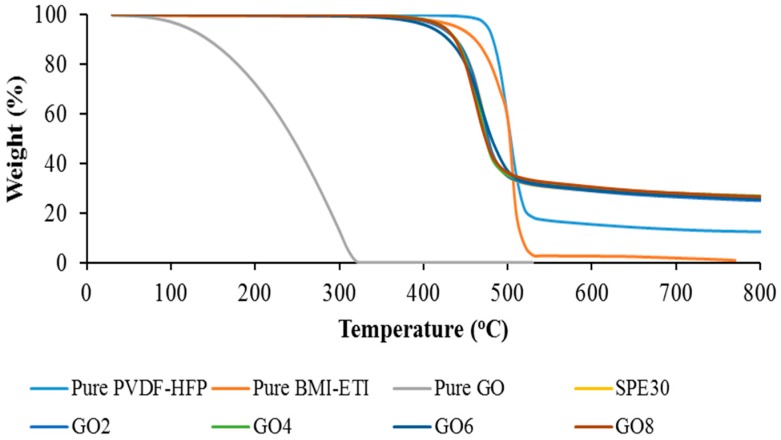
TGA thermograms for pure P(VdF-HFP), pure EMI-BTI, pure GO, SPE30 and different weight percentages of GO-doped PVdF-HFP-[EMI-BTI]-based SPE samples.

TGA thermograms for pure P(VdF-HFP), pure EMI-BTI, pure GO, SPE30 and the different weight percentage of GO-doped SPEs are shown in Figure 7. Based on the figure, the SPEs are thermally stable up to around 440 °C and this thermal durability is desirable for most electrochemical device applications. As seen in Table 4, the initial addition of ionic liquid was found to decrease the decomposition temperature of the SPE from 501.28 °C to 448.96 °C. This may be due to the complexation of ionic liquid with the P(VdF-HFP) which has been demonstrated in the XRD studies that decrease the crystallinity of the SPE and resulting in lower thermal stability.

As seen in the figure, pure GO was found to be decomposing at a temperature around 194 °C because of the decomposition of the oxygen functional group and totally decomposed at 320 °C. Graphene oxide normally exfoliates and decomposes at high temperatures to form finely dispersed amorphous carbon [22]. However, as seen in the figure in the GO-doped SPE samples, there is no trace of GO decomposing at the temperature around 194–320 °C. This phenomenon shows that there are complexations of the GO and the SPE occurring in the polymer matrix and this is in agreement with the XRD studies. It is also found that the addition of GO only slightly decreases the decomposition temperature of the SPE. As seen in the table, the decomposition temperature of SPE30 that was at 448.96 °C decreases to 448.76 °C, 444.53 °C, 438.95 °C and 437.84 °C for GO2 to GO8, respectively. This shows that the addition of GO only slightly decreases the thermal stability of the SPE by a negligible value.

### 3.6. Electrochemical Impedance (EIS) Performance of the EDLC

The electrochemical performance of AC/GO-doped SPE/AC supercapacitors were evaluated using CV analysis. Cyclic voltammograms were performed for GO2, GO4, GO6 and GO8 with a potential window from −1 to 1 V at 5, 10, 20, 30, 40, and 50 mV/s scan rates.

**Figure 8:**
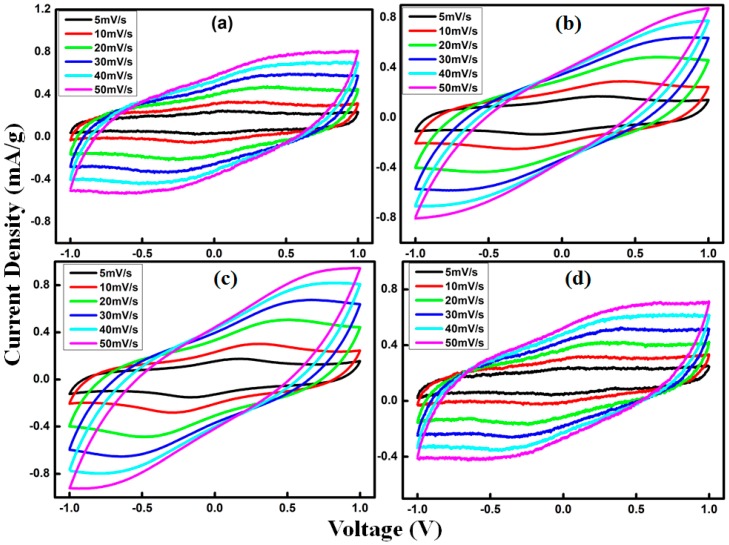
Variation in specific capacitance at different scan rates for EDLCs fabricated with sample (**a**) GO2; (**b**) GO4; (**c**) GO6; (**d**) GO8.

**Figure 9:**
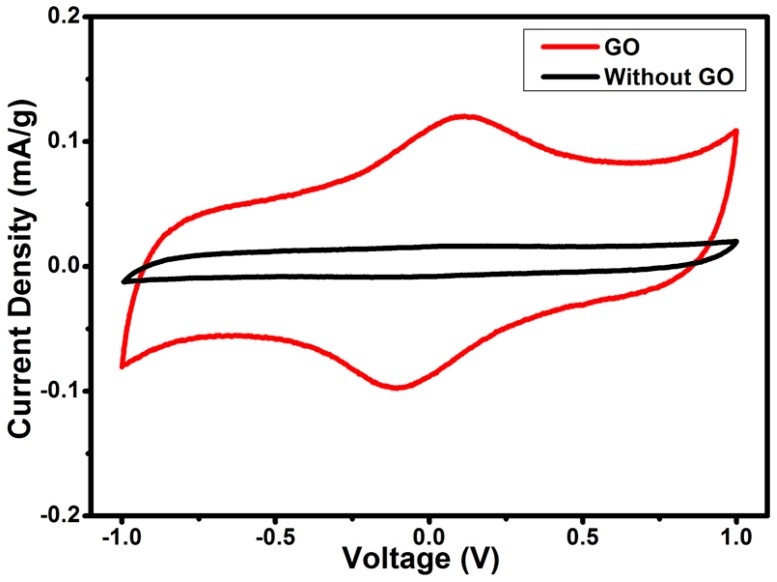
CV curve for EDLC fabricated with SPE with (GO6) and without (SPE30) GO at scan rate of 3 mV/s.

Using electrode mass, the currents measured were normalized. The specific capacitance *C_sp_* can be obtained using the equation below:

(3) $$\;C_{sp}=\frac{It}{MvV}$$

where *It* is the area of the CV curve, *m* is the mass of active material, *v* is the scan rates and *V* is a potential window. Figure 8 shows the CV curves of GO2, GO4, GO6 and GO8 at different scan rates, respectively. CV curves that have rectangular shapes exhibited excellent electrochemical stability, especially at the higher scan rate. Figure 9 shows a rectangular CV, which is nearly mirror image symmetry of the current responses to zero line. This confirms the electric double layer capacitive behavior [37]. In addition, small reversible humps are also observed (at a scan rate of 3 mV/s), revealing that redox reactions are also contributing to the specific capacitance. These small humps are arising from the fast switching of Na^+^ in the pores of activated carbon electrodes. In fact, electronegative functional groups on GO sheets served as channels for rapid switching of Na^+^ by interacting with the ions. However, in the case of cells using GPE without GO, redox peaks were not prominent, confirming that in the absence of GO, intercalation of Na^+^ did not prevail. Similar results were found by G.P. Pandey *et al.* [38,39]. The calculated specific capacitance of GO2, GO4, GO6 and GO8 were 20 F/g, 24 F/g, 29.6 F/g and 24 F/g at a scan rate of 3 mV/s respectively.

**Figure 10:**
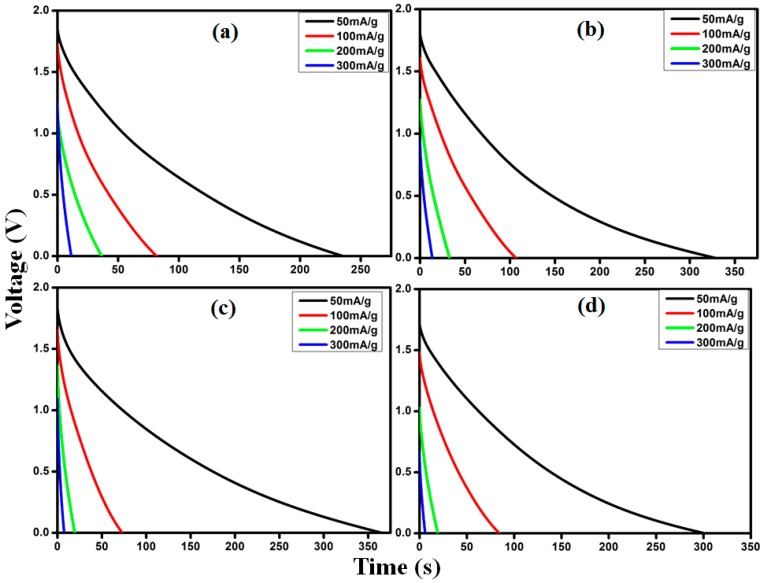
Galvanostatic discharge curves for EDLCs fabricated with sample (**a**) GO2; (**b**) GO4; (**c**) GO6; (**d**) GO8.

The cyclic discharge can further evaluate the electrochemical capacitance of materials. Figure 10 shows the galvanostatic discharge curves for the assembled supercapacitor cells at current densities of 50, 100, 200, 300 mA/g at room temperature for the GO doped SPEs. It is evident from the discharge curves that assembled cells exhibit linear characteristics that represent outstanding electrochemical performance from the supercapacitor [40].

**Figure 11:**
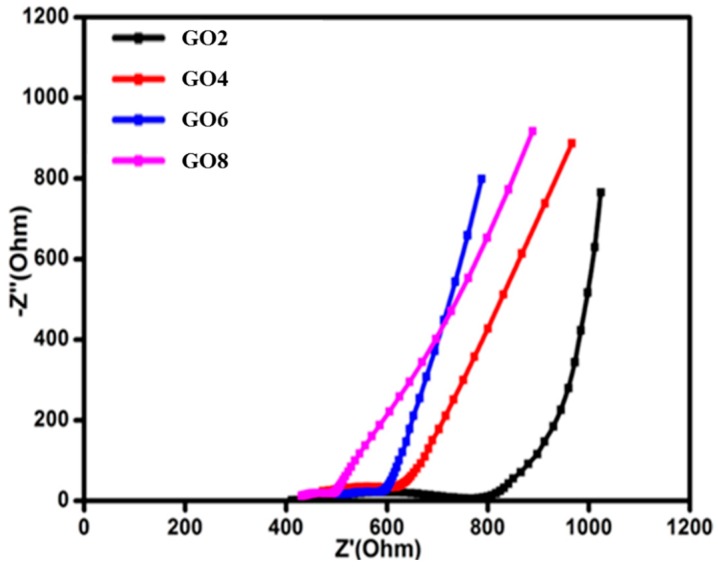
Nyquist Plots for EDLCs fabricated with PVdF-HFP-[EMI-BTI]-based SPE samples with different weight percentages of GO.

For further explanation of the high electrochemical performance, an electrochemical impedance spectrum (EIS) was used to examine GO-doped SPE-based EDLCs. The equivalent series resistance (ESR) could be obtained from the intersection of the curves with the axis of real impedance. The difference in the ESRs of supercapacitor cells is attributed to the different conductances of electrode materials [41]. It is evident from Figure 11 that the ESR of the GO6 was found to be minimal as compared to GO4 and GO8. This also confirms the high conductive and good electrochemical performance of the EDLC fabricated with GO6.

**Figure 12:**
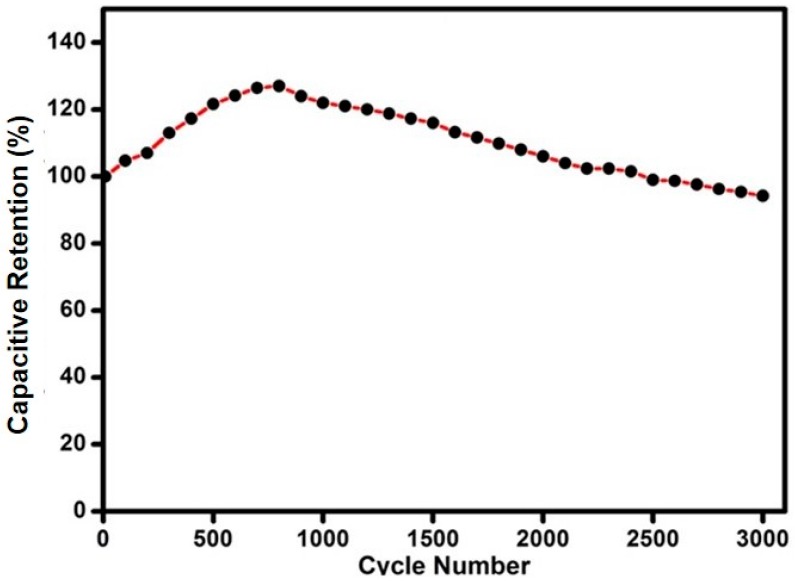
The variation of specific capacitance as a function of cycle number from 5th to 3000th for GO6.

Long-term cycling stability test was a key factor to evaluate the SPE performance for practical applications. The long-term cycling performance over 3000 cycles of GO6 was analyzed by repeating the charge/discharge test at a current density of 200 mA/g and the results are shown in Figure 12. Initially, the specific capacitance increased at the start of charge–discharge cycling. This increment in specific capacitance was due to the activation of carbon electrodes on charge–discharge cycling. When all electrode sites are activated, specific capacitance reached its peak until 800 cycles. However, with further charge–discharge cycling, specific capacitance decreased slowly. After charge–discharge cycling of 3000 cycles, the capacitive retention dropped by only 6%. Our results are comparable to the already reported results summarized in Table 5. This confirmed the excellent stability of GO-based SPEs for energy storage applications. Therefore, these results indicate that GO6 is a promising candidate for high-performance energy storage systems.

## 4. Conclusions

In this work, solid polymer electrolyte (SPE) films composed of semi-crystalline P(VdF-HFP) copolymer as the host polymer and EMI-BTI as the ionic liquid were synthesized, and their performances were evaluated for electrical double layer capacitors (EDLC). The addition of graphene oxide as filler does help in enhancing the ionic conductivity of the solid polymer electrolyte. The conductivity of the electrolyte increases with the weight percentage of graphene oxide. The highest conductivity obtained was 12.25 μS cm^−1^ for GO8. A temperature dependence study of the GO-doped SPE showed that the ionic conduction mechanism follows Arrhenius. These SPEs are thermally stable up to 400 °C with the addition of ionic liquid-formed complexation with the polymer, while GO addition only decreases the decomposition temperature slightly. The electrochemical performance studies of GO-doped SPE supercapacitors showed that GO6 containing 6% of GO had a maximum specific capacitance of 29.6 F/g at a scan rate of 3 mV s^−1^ compared to other SPEs due to high conductivity and effective ion transfer. Moreover, GO6 SPE-based EDLCs exhibited an excellent cyclic retention of 94% even after 3000 cycles. These results suggest that GO-doped SPE can play a vital role in energy storage applications.

## Figures and Tables

**Table 1:** The composition of PVdF-HFP-[EMI-BTI]-based SPE samples.

Electrolytes	Composition PVdF-HFP:EMI-BTI:GO	PVdF-HFP (g)	EMI-BTI (g)	GO (g)
SPE10	90:10:0	0.900	0.100	-
SPE20	80:20:0	0.800	0.200	-
SPE30	70:30:0	0.700	0.300	-
SPE40	60:40:0	0.600	0.400	-
GO2	68.6:29.4:2	0.686	0.294	0.02
GO4	67.2:28.8:4	0.672	0.288	0.04
GO6	65.8:28.2:6	0.658	0.282	0.06
GO8	64.4:27.6:8	0.644	0.276	0.08

**Table 2:** Ionic conductivity and activation energy values of the PVdF-HFP-[EMI-BTI]-based SPE samples at room temperature.

Electrolytes	Ionic Conductivity (µS cm^−1^)	Activation Energy, *E*_a_ (eV)
SPE10	2.09	0.147
SPE20	3.11	0.120
SPE30	3.92	0.104
SPE40	1.31	0.171
GO2	5.03	0.095
GO4	5.88	0.089
GO6	7.40	0.079
GO8	12.25	0.076

**Table 3:** Possible assignments of some significant peaks in FTIR spectra of pure PVdF-HFP and EMI-BTI.

Samples	Wavenumber (cm^−1^)	Possible Assignments
Pure PVdF-HFP	762, 854	α-phase
	796	CF_3_ stretching
	838, 875	Amorphous region
	1062	C-C skeletal vibration
	1182	Symmetrical stretching of -CF_3_
	1202	Asymmetrical stretching of -CF_2_-
	1404	-C-F-strectching
Pure EMI-BTI	854	In-plane bending of imidazole ring
	949	In-plane bending of CNC
	1352	Symmetrical bending in-plane of CH
	1386	In-plane asymmetrical stretching of imidazole ring
	1457	Symmetrical in-play bending of imidazole ring
	1576	HCN deformation
	2980	CH stretching in methyl group
	3120, 3157	=C-H stretching
	3587	CH stretching

**Table 4:** Decomposition temperature for PVdF-HFP-[EMI-BTI]-based SPE samples.

Samples	Decomposition Temperature, *T*_D_ (°C)
Pure PVdF-HFP	501.28
Pure EMI-BTI	500.15
Pure GO	194.08
SPE30	448.96
GO2	448.78
GO4	444.53
GO6	438.95
GO8	437.84

**Table 5:** Summary of reported results similar to this work.

Electrolyte Material	Electrodes	Current Density (A/g)	Total Cycles	Capacitive Retention (%)	Reference
PVA–H2SO4	Activated carbon	Nil	3000	90.77	[42]
HQ-H2SO4	Activated carbon	Nil	4000	65	[43]
PVDF-HFP+GO	Activated carbon	0.2	3000	94	This work
